# Supplementary figures and images for: Slow sink rate in floated-demersal longline and implications for seabird bycatch risk
Source: PLoS One. 2022 Apr 28;17(4):e0267169. doi: 10.1371/journal.pone.0267169 (PMC9049334; doi:10.1371/journal.pone.0267169)

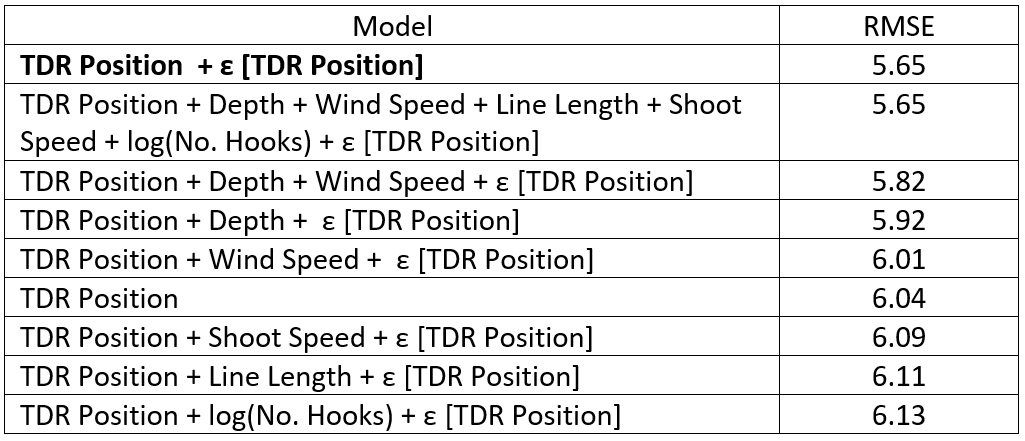

Supplement: S1 Table — Table shows model tested and corresponding Root Mean Square Error (RMSE) calculated using Leave-One-Out Cross-Validation (LOO-CV). Best performing model highlighted in bold. ε [TDR Position] denotes a model in which separate estimates of the residual variance were made for each Position. (PNG) [file pone.0267169.s001.png]

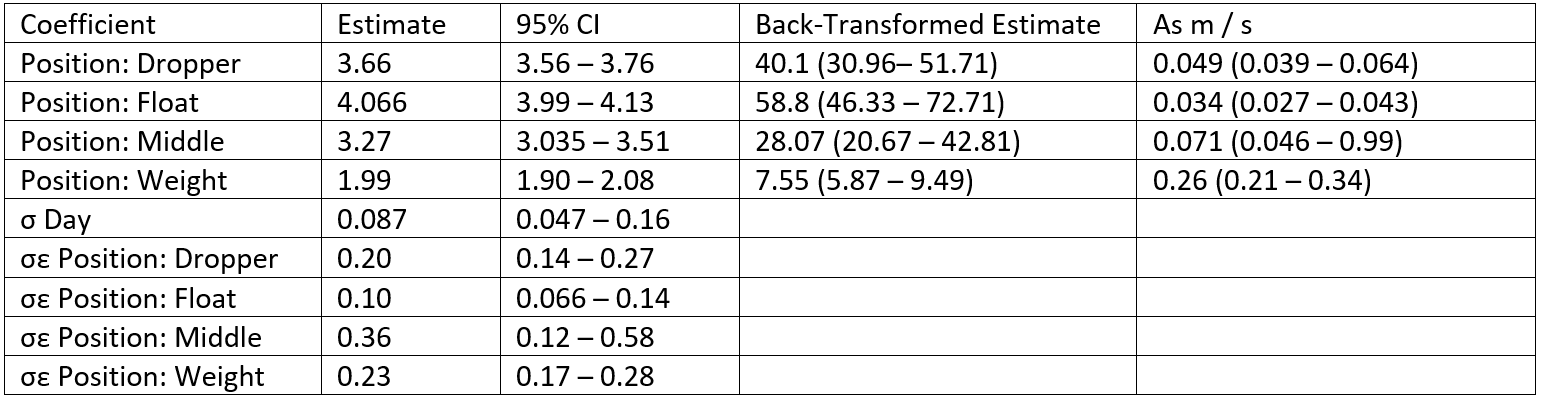

Supplement: S2 Table — Table display coefficients from a model in which time was modelled using a log transformation. Back-transformed coefficients for sink speed also displayed in original units as well as expressed as the average sink rate (m / s) from 0–2 metre depth. σ Day is the random effect associated with day on which longlines were deployed. σε is the residual variation in the model–note that different residual variation parameters were estimated for each location in this model. n = 91 observations, 11 days. Model marginal R2 = 0.93 (95% CRI: 0.89–0.95); Model conditional R2 = 0.94 (0.91–0.96). (PNG) [file pone.0267169.s002.png]

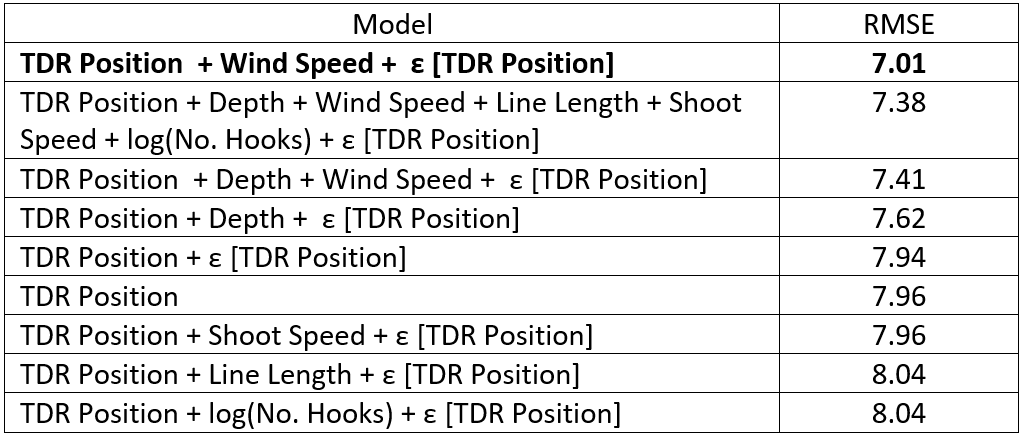

Supplement: S3 Table — Table shows model tested and corresponding Root Mean Square Error (RMSE) calculated using Leave-One-Out Cross-Validation (LOO-CV). Best performing model highlighted in bold. ε [TDR Position] denotes a model in which separate estimates of the residual variance were made for each Position. (PNG) [file pone.0267169.s003.png]

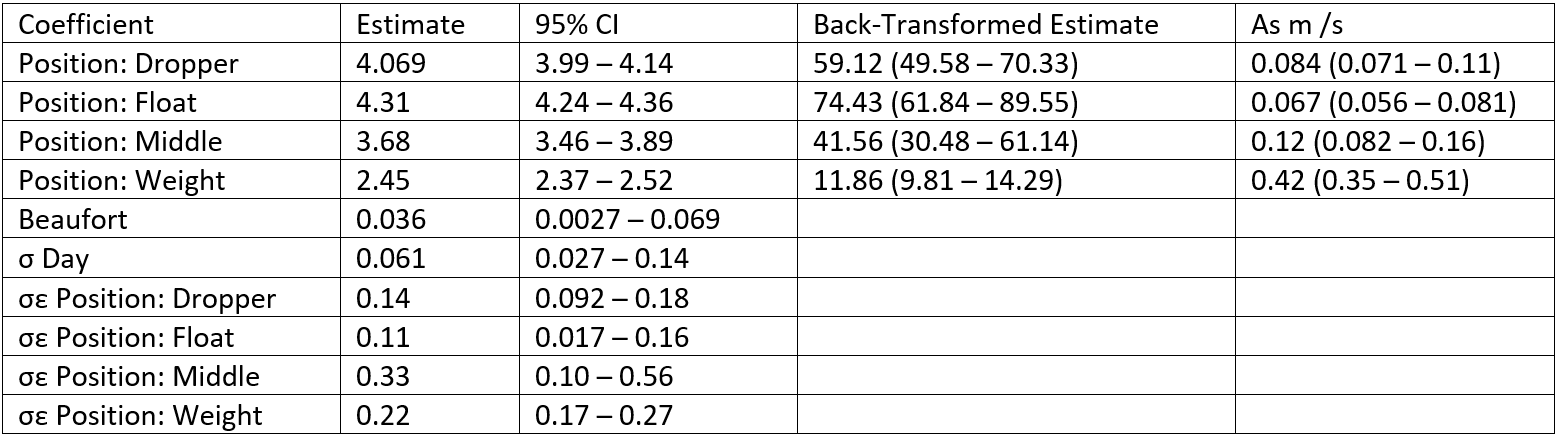

Supplement: S4 Table — Table display coefficients from a model in which time was modelled using a log transformation. Back-transformed coefficients for sink speed also displayed in original units as well as expressed as the average sink rate (m / s) from 0–5 metre depth. Back-transformed estimates assume Beaufort scale is set at its modal value. σ Day is the random effect associated with day on which longlines were deployed. σε is the residual variation in the model–note that different residual variation parameters were estimated for each location in this model. n = 91 observations, 11 days. Model marginal R2 = 0.93 (95% CRI: 0.89–0.95); Model conditional R2 = 0.94 (0.90–0.96). (PNG) [file pone.0267169.s004.png]

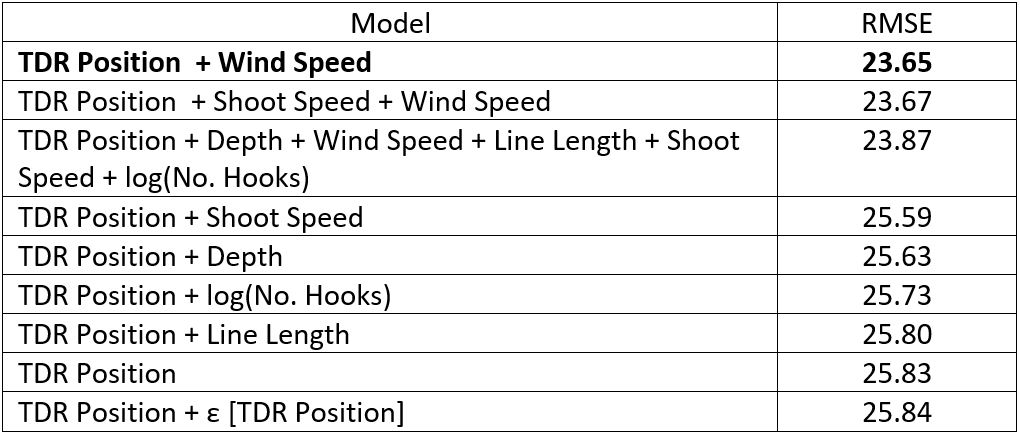

Supplement: S5 Table — Table shows model tested and corresponding Root Mean Square Error (RMSE) calculated using Leave-One-Out Cross-Validation (LOO-CV). Best performing model highlighted in bold. ε [TDR Position] denotes a model in which separate estimates of the residual variance were made for each Position. (PNG) [file pone.0267169.s005.png]

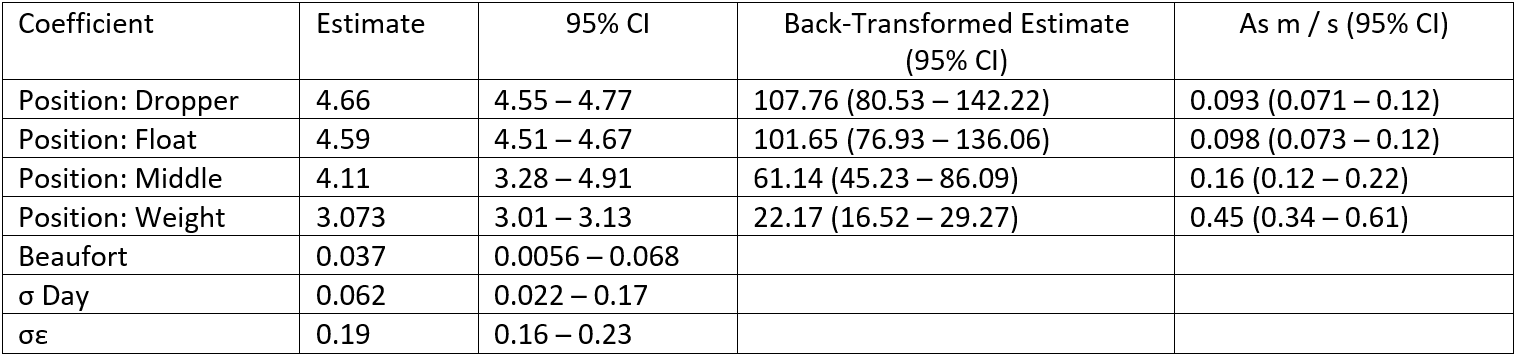

Supplement: S6 Table — Table display coefficients from a model in which time was modelled using a log transformation. Back-transformed coefficients for sink speed also displayed in original units as well as expressed as the average sink rate (m / s) from 0–10 metre depth. Back-transformed estimates assume Beaufort scale is set at its modal value σ Day is the random effect associated with day on which longlines were deployed. σε is the residual variation in the model. n = 91 observations, 11 days. Model marginal R2 = 0.92 (95% CRI: 0.88–0.94); Model conditional R2 = 0.93 (0.91–0.95). (PNG) [file pone.0267169.s006.png]

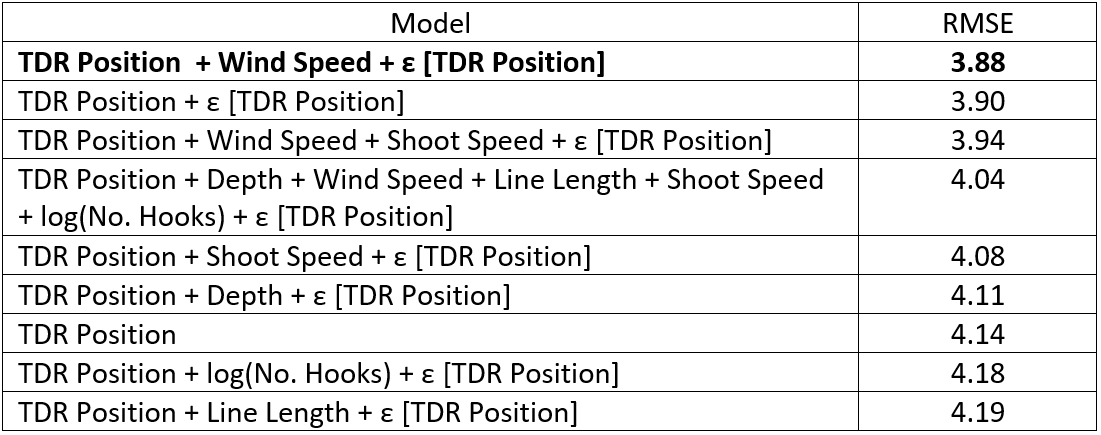

Supplement: S7 Table — Table shows model tested and corresponding Root Mean Square Error (RMSE) calculated using Leave-One-Out Cross-Validation (LOO-CV). Best performing model highlighted in bold. ε [TDR Position] denotes a model in which separate estimates of the residual variance were made for each Position. (PNG) [file pone.0267169.s007.png]

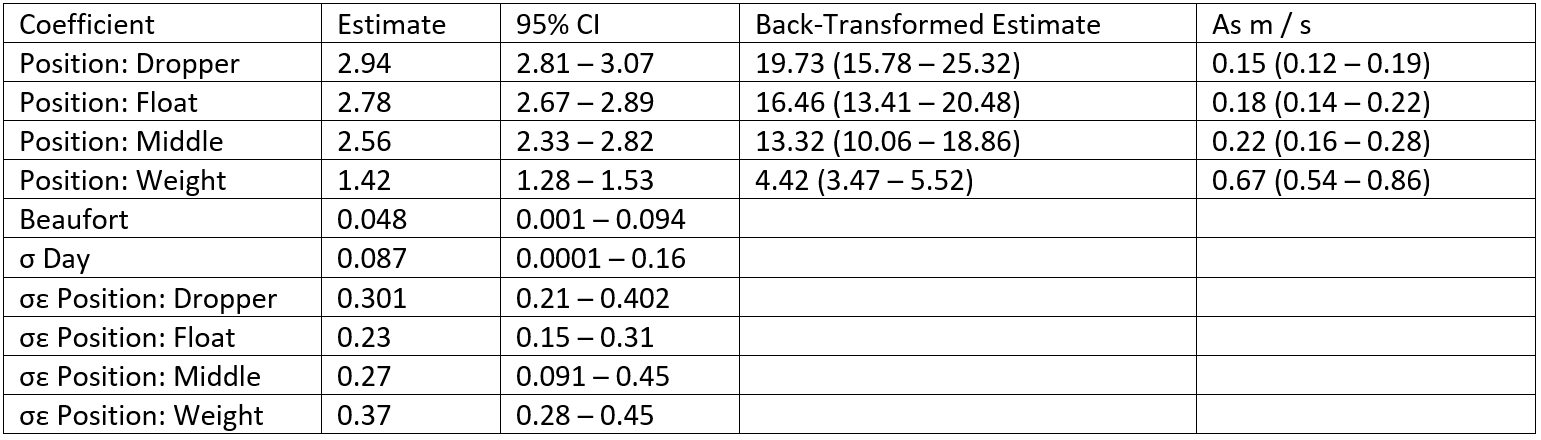

Supplement: S8 Table — Table display coefficients from a model in which time was modelled using a log transformation. Back-transformed coefficients for sink speed also displayed in original units as well as expressed as the average sink rate (m / s) from 2–5 metre depth. Back-transformed estimates assume Beaufort scale is set at its modal value. σ Day is the random effect associated with day on which nets were deployed. σε is the residual variation in the model–note that different residual variation parameters were estimated for each net location in this model. n = 91 observations, 11 days. Model marginal R2 = 0.84 (95% CRI: 0.72–0.91); Model conditional R2 = 0.86 (0.74–0.92). (PNG) [file pone.0267169.s008.png]

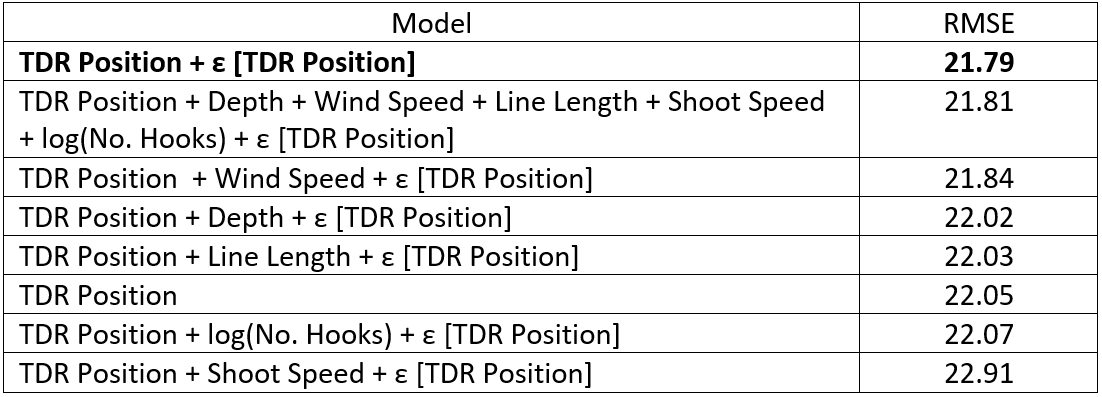

Supplement: S9 Table — Table shows model tested and corresponding Root Mean Square Error (RMSE) calculated using Leave-One-Out Cross-Validation (LOO-CV). Best performing model highlighted in bold. ε [TDR Position] denotes a model in which separate estimates of the residual variance were made for each Position. (PNG) [file pone.0267169.s009.png]

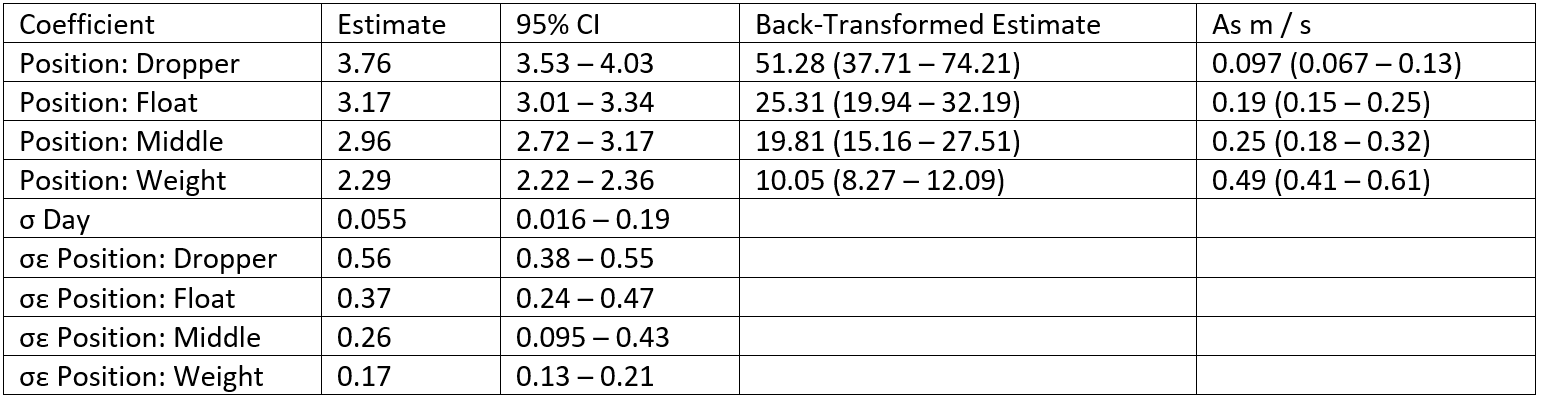

Supplement: S10 Table — Table display coefficients from a model in which time was modelled using a log transformation. Back-transformed coefficients for sink speed also displayed in original units as well as expressed as the average sink rate (m / s) from 5–10 metre depth. σ Day is the random effect associated with day on which longlines were deployed. σε is the residual variation in the model–note that different residual variation parameters were estimated for each location in this model. n = 91 observations, 11 days. Model marginal R2 = 0.77 (95% CRI: 0.68–0.82); Model conditional R2 = 0.79 (0.69–0.83). (PNG) [file pone.0267169.s010.png]

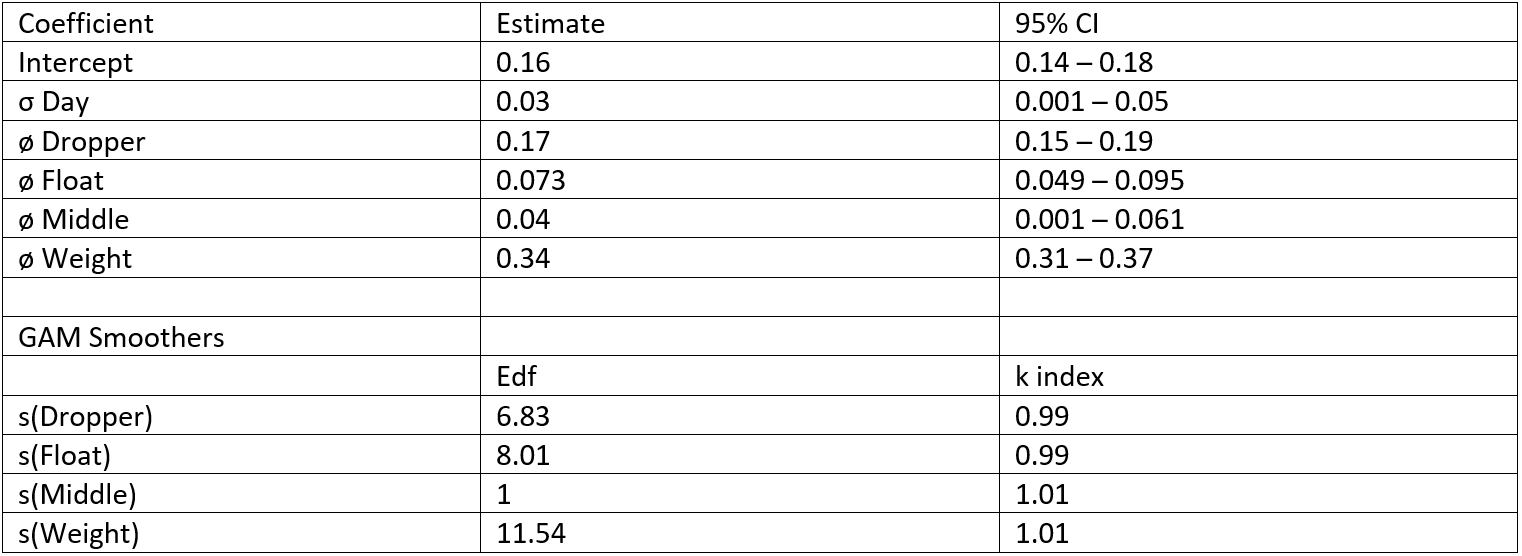

Supplement: S11 Table — σ Day represents the random effect associated with longlines deployed on different days. The temporal autocorrelation in depth over time is represented as ø and was estimated separately for each gear location. Different smoothers were fitted for each gear location and details on these smoothers (estimated degrees of freedom and k index are also displayed). Model R2 = 0.88. (PNG) [file pone.0267169.s011.png]

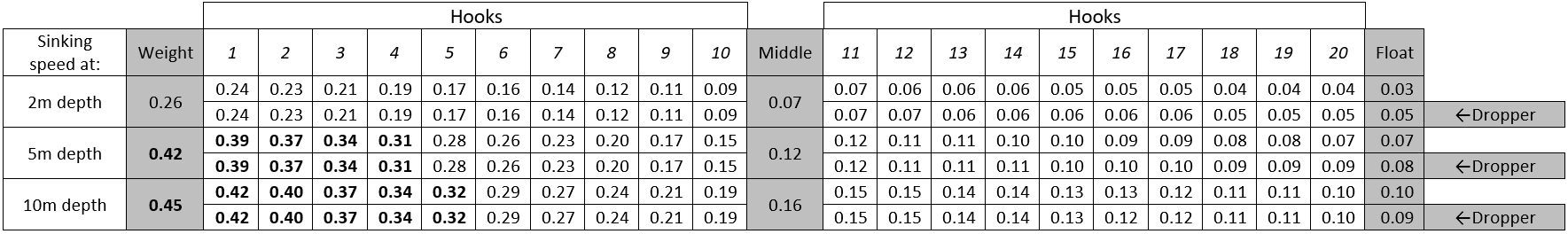

Supplement: S12 Table — Using a linear series from TDRs recordings at different positions of the gear (in grey shading). In bold are rates equal or over ACAP recommendation (0.3 m/s). (PNG) [file pone.0267169.s012.png]

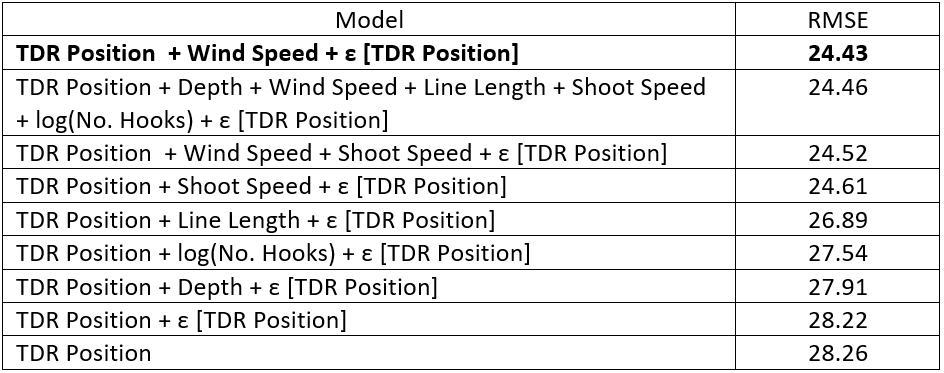

Supplement: S13 Table — Table shows model tested and corresponding Root Mean Square Error (RMSE) calculated using Leave-One-Out Cross-Validation (LOO-CV). Best performing model highlighted in bold. ε [TDR Position] denotes a model in which separate estimates of the residual variance were made for each Position. (PNG) [file pone.0267169.s013.png]

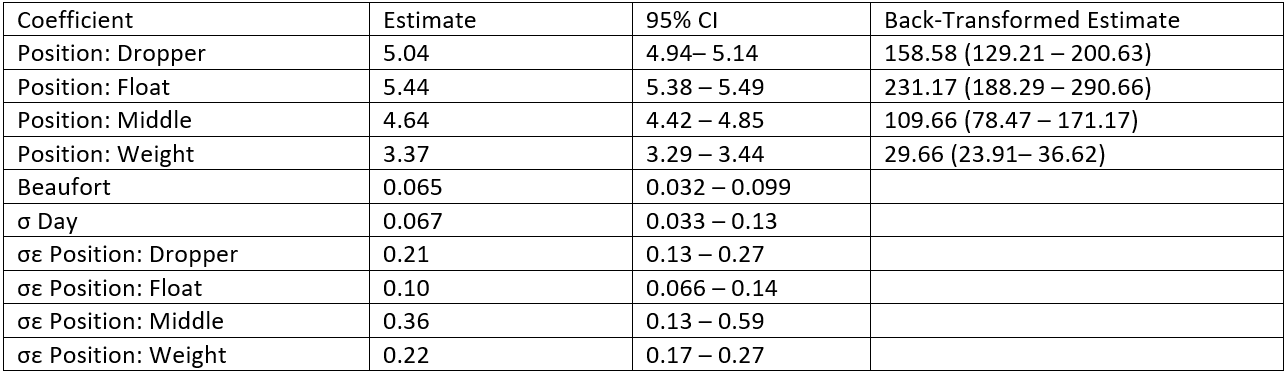

Supplement: S14 Table — Table display coefficients from a model in which distance from stern was modelled using a log transformation. Back-transformed coefficients for distance travelled also displayed in original units. Back-transformed estimates assume Beaufort scale is set at its modal value. σ Day is the random effect associated with day on which longlines were deployed. σε is the residual variation in the model–note that different residual variation parameters were estimated for each location in this model. n = 91 observations, 11 days. Model marginal R2 = 0.93 (95% CRI: 0.91–0.95); Model conditional R2 = 0.95 (0.94–0.96). (PNG) [file pone.0267169.s014.png]

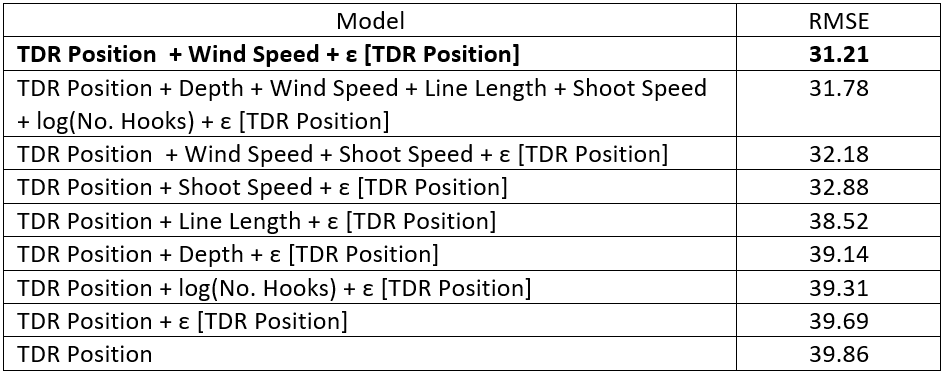

Supplement: S15 Table — Table shows model tested and corresponding Root Mean Square Error (RMSE) calculated using Leave-One-Out Cross-Validation (LOO-CV). Best performing model highlighted in bold. ε [TDR Position] denotes a model in which separate estimates of the residual variance were made for each Position. (PNG) [file pone.0267169.s015.png]

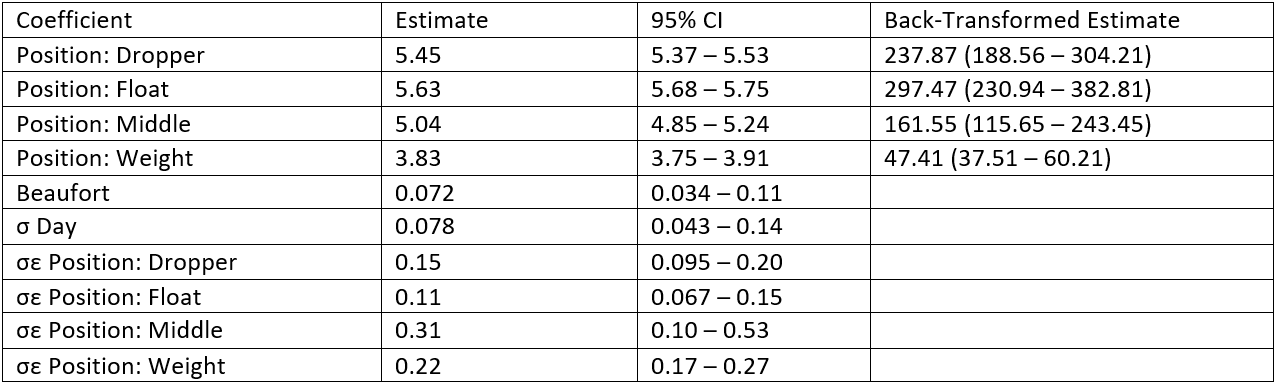

Supplement: S16 Table — Table display coefficients from a model in which distance from stern was modelled using a log transformation. Back-transformed coefficients for distance travelled also displayed in original units. Back-transformed estimates assume Beaufort scale is set at its model value. σ Day is the random effect associated with day on which longlines were deployed. σε is the residual variation in the model–note that different residual variation parameters were estimated for each location in this model. n = 91 observations, 11 days. Model marginal R2 = 0.92 (95% CRI: 0.89–0.95); Model conditional R2 = 0.95 (0.93–0.96). (PNG) [file pone.0267169.s016.png]

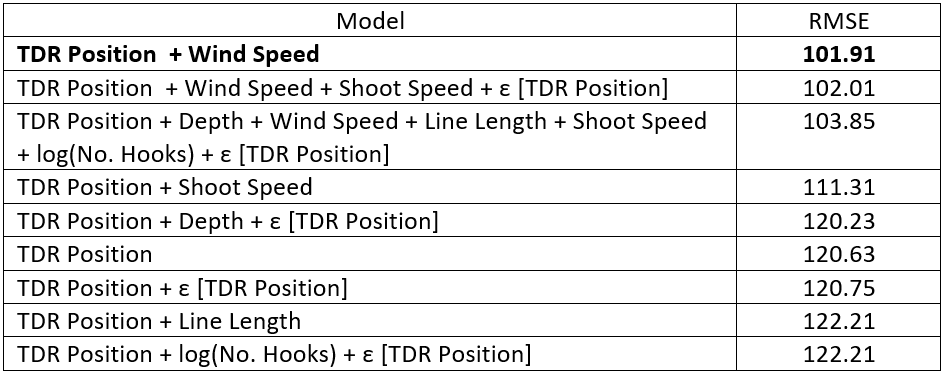

Supplement: S17 Table — Table shows model tested and corresponding Root Mean Square Error (RMSE) calculated using Leave-One-Out Cross-Validation (LOO-CV). Best performing model highlighted in bold. ε [TDR Position] denotes a model in which separate estimates of the residual variance were made for each Position. (PNG) [file pone.0267169.s017.png]

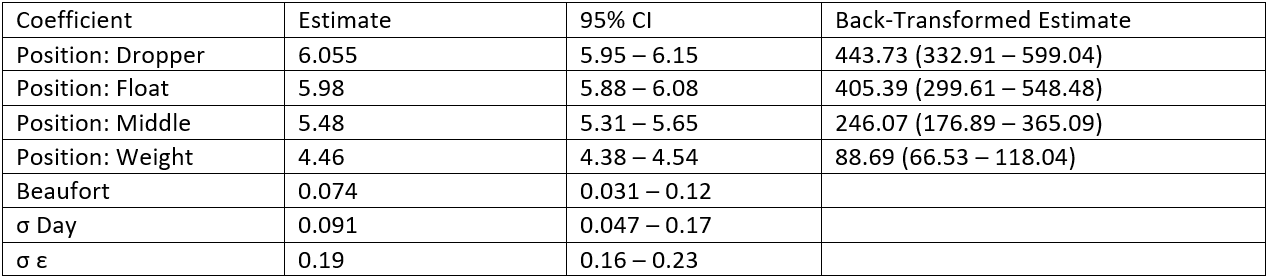

Supplement: S18 Table — Table display coefficients from a model in which distance from stern was modelled using a log transformation. Back-transformed coefficients for distance travelled also displayed in original units. Back-transformed estimates assume Beaufort scale is set at its modal value. σ Day is the random effect associated with day on which longline were deployed. σε is the residual variation in the model. n = 91 observations, 11 days. Model marginal R2 = 0.91 (95% CRI: 0.81–0.94); Model conditional R2 = 0.94 (0.91–0.96). (PNG) [file pone.0267169.s018.png]
