# Supplementary material for: Slow sink rate in floated-demersal longline and implications for seabird bycatch risk
Source: PLoS One. 2022 Apr 28;17(4):e0267169. doi: 10.1371/journal.pone.0267169 (PMC9049334; doi:10.1371/journal.pone.0267169)
Supplement: Attachment — Submitted filename: Response to Reviewers_Rouxel et al..docx [file pone.0267169.s019.docx]

Response to reviewers:

| **Reviewer 1 - Comments** | **Authors response** |
| --- | --- |
| In this study, the authors collected and analyzed sink rate data from a floated demersal longliner in the Eastern North Atlantic. In my opinion, this research topic is important for seabird bycatch mitigation. For example, see Dietrich et al, 2008. The study design is appropriate, although there are several places detailed below that needs to be further clarified. The statistical analysis as used in this manuscript is not satisfactory, and both the analysis and the interpretation of the results can be further improved.  In this study, the use of spline is good as it smoothes out the noise and it is also straight forward to calculate the sink rate, which is the 1st derivative of the spline. Both Fig5a and 5b look good. However, Fig5c and 5d look problematic. First, the interval estimates on the right-hand side are way too wide. Second, most data tracks stop midway. Either the depth readings didn't change afterwards or the device stopped recording at certain depth. I think the authors just chopped the second half of the data if depth readings remained stable. This is understandable, but the authors need to document it in the methods section. Assuming this is the case, the use of spline smoothing is not appropriate for the second half of the data in Fig5c and d due to this data cutting. For example, in Fig5c, to the right of ~110 on the x-axis, the spline flattens while none of the tracks flattens, and the flattening is simply due to inappropriate smoothing across multiple chopped tracks. The authors need to be careful here. Even though this portion of the data (>10m) has little practical significance to this study, both the methods and the interpretation of the result need to be correct.  In addition, what is the observed target catch and bycatch rates for these experimental trials? If the authors plan to save these statistics for another paper, at least provide a summary here. | We agree that data on fish catch and bycatch rate would have been valuable. However, due to the nature of the project, we chose not to collect those data during the field work. With only one person on board to calibrate, deploy and retrieve several TDRs for each fishing operation during the fishing trip, systematic data collection of fish catch and bycatch rate would have been too demanding to the worker (setting/retrieving a longline takes several hours). Collecting fish catch data would have been useful if we were testing an alternative longline configuration (paired trial analysis), which was not the objective of this study, however. This will be important however in the future whilst testing mitigation measures.  We agree that model selection tables should have been included and are now within the Supplementary Material / Appendix.  Regarding figs 5, we processed the dataset prior to the statistical analysis, keeping only the TDR recordings up to ~25 meters depth. The idea was to give us enough “buffer” information to analyse the 0-10m sinking profiles. We decided not to include in the analysis the recording which exceeded those depths, since we were only interested in the upper portion of the water column that are most at risk to seabird bycatch. Data points past this threshold were also recorded but didn’t provide valuable information for the bycatch focused analysis, and consistency of TDRs settings were not ensured past those points (e.g. some TDRs stopped recording before reaching the seabed, some at the maximum depths and others even recorded the hauling phase).  Figs 5a and 5b were plotted within the ~120 seconds of deployment, which encompass the “end” of the riskiest bycatch depths (up to 10m). In line with the reviewer’s suggestion, we have re-plotted Figs 5c and 5d over a more restricted time-period determined by the time at which the first TDR deployment within that gear position stopped recording (this is now specified in the figure legend). Hopefully this ensures that the splines we show time periods with sufficient data. We are also clearer in the methods that TDR depth recording was truncated once a depth of 25 m was attained. We also changed the figure legend for figure 5 accordingly so it now reads:  ‘Figure 5 –The relationship between TDR depth and time elapsed since line was set at each TDR location along the longline. In figures 5a and 5b estimated sink rate is displayed over the first 2 minutes (120 seconds) of deployment. In figures 5c and 5d the x-axis is limited to the time at which the first deployment in set stopped recording upon reaching 20-25 metres of depth, which occurred before 2 minutes had elapsed. The solid line represents the average relationship across all recorded TDRs deployed at a specific location estimated via a GAMM. The solid line is red when predicted sink rate is below 0.3 m/s and black when predicted sink rate is above 0.3 m / s. The blue poylgon around the line encompasses the 95% confidence interval (95% CI) around model predictions. The raw data from each TDR deployment is also displayed as a grey line to highlight variation between different longline deployments on different days. The dashed diagonal line on the plot represents the trajectory that would be achieved by a hypothetical TDR sinking at a constant rate of 0.3 m / s.’ |
| The model selection results are missing. Add a table with the loo score for each candidate model. | We agree that model selection tables should have been included and are now within the Supplementary Material / Appendix. |
| line 127: even though there is no standard configuration, the authors need to provide evidence that the configuration presented here is representative of the actual situation. | Sources are from personal communications with skippers and experienced fishery observer. Other than direct observations and communications with the stakeholders, there is - to the best of our knowledge - no published literature describing the configuration of this specific fishing gear. We have however added 3 new references of studies that described similar semi-pelagic (floated-demersal) longline fisheries’ configuration while targeting Hake across the Atlantic.   - Erzini, K., Gonçalves, J. M. S., Bentes, L., Lino, P. G., & Ribeiro, J. (2001). The hake deepwater semi-pelagic (“pedra-bola”) longline fishery in the Algarve (southern Portugal). Fisheries Research, 51(2-3), 327-336. - Paterson, J. R., Yates, O., Holtzhausen, H., Reid, T., Shimooshili, K., Yates, S., ... & Wanless, R. M. (2019). Seabird mortality in the Namibian demersal longline fishery and recommendations for best practice mitigation measures. Oryx, 53(2), 300-309. - Petersen, S. L., Honig, M. B., Ryan, P. G., & Underhill, L. G. (2008). Line sink rates and implications for seabirds, target and non-target fish species in the south African demersal longline fishery. Understanding and Mitigating Vulnerable Bycatch in southern African Trawl and Longline Fisheries, 191, 190. |
| line 180: Where is your settings for the priors, which is an essential component of every Bayesian model? | The information on settings for priors and MCMC settings now added in methods:  ‘For all the fixed effect predictors in our model we used normal priors with a mean of 0 and a variance of 100. Priors for variance components were set using an inverse-Wishart distribution. For a single variance component, the inverse-Wishart distribution is dictated by two parameters, *V* and *v*, in MCMCglmm notation following Hadfield (2010). Hadfield (2010) refers to ν as the ‘degree of belief parameter’ and smaller values of *v* denote weaker belief in prior values for the unknown variance parameter V. We set v = 0.002 to create a diffuse prior for unknown variance components and ran 3 MCMC chains that began at dispersed values for V (0.05, 0.5, and 1.0 respectively, with v fixed at 0.002). Convergence of chains was examined through inspection of trace plots and calculation of the Gelman-Rubin statistic (Gelman & Rubin 1992). Chains were run for 20000 rounds, with a burn-in of 3000 rounds and a thinning interval of 10 to ensure each parameter had an effective sample between 1000 – 2000 per MCMC chain.’  Gelman, A., & Rubin, D. B. (1992). Inference from iterative simulation using multiple sequences. *Statistical science*, *7*(4), 457-472.   - Hadfield, J. D. (2010). MCMC methods for multi-response generalized linear mixed models: the MCMCglmm R package. *Journal of statistical software*, *33*, 1-22. |
| line 181: which type of CI did you pick, symmetric or HDI? | We specified that these are equal-tail (i.e. symmetric) intervals: ‘When reporting the results from our Bayesian analyses we report parameter estimates (posterior means) and corresponding equal-tail 95% credibility interval (CRI) values.’ |
| line 203: Are these 97 trials conducted on the same fishing vessel with the help of a single observer? This is important because otherwise "observer id" and "vessel id" would be two important factors affecting the sink rate and they should be incorporated into the model. | The trials were conducted on a single vessel and monitored by the same fishery observer. In Field work methodology, we have added the following sentence *“All data were collected by a single observer and onboard the same vessel”*. |
| line 262: Rephrase this sentence. I can guess what you are trying to say by looking at the table in the Appendix. Also, it is calculation not simulation. | Answer: we changed “simulation” to “calculation”. |
| Fig5a: Looking at this figure, I can see that either the device used in this study has a low depth resolution or the data have been truncated. State in the method section what is the design accuracy of this device and the resolution of the recorded data. The surface layer is the most important in this study. What is the design accuracy of this device for the surface layer? Did you calibrate the device (pressure sensor) before using it or after the trials? Readings may drift under harsh environmental conditions. A seperate section on the validity of this device and its readings would further strenghen this study. | In the Methodology section we stated that “Each TDR was calibrated with the Dive Logging option, using wet/dry sensors (activation by entry into the water) and 2Hz log rate (recording every 0.5 sec), as well as 12-bit Data Points defined resolution for the Pressure sensor.”  To better describe design accuracy, we have also added in the Methodology section “TDRs were calibrated with 50 bar sensors, providing a pressure resolution better than 15cm with the above setting.”  Given the short-in-time nature of the deployments data (up to 25m), we consider the potential for drift in pressure to be more of an issue in longer deployments, and minimal in our case.  As stated above, the devices continued to record depths past 25m, but we only conducted analysis on a database made of the 0 to 25m recordings. Given the focus of this paper being on bycatch risks within surface waters, we considered these extra data points being out of scope for our paper’s focus. |
| line 45: Do not capitalize the second part of a scientific name. | corrected |
| line 49: Use different dashes consistently | corrected |
| line 64: What do you mean here? "lofted off the seabird"? | it was a typo error; changed to “seabed” |
| line 179: not grammatically correct. | Re-worded as: ‘Here we report marginal estimates by performing back-transformation over the random effects distribution in our models. Marginal estimates of time to depth were then converted to estimates of sink rate (m / s).’ |
| line 240: typo | Answer: corrected |
| Fig2: which base map did you use, where is the attribution, what projection did you use? Instead of a star, I think a rectangle shape is more appropriate. Also, it might be better to use a zoomed-in main map and keep this one as an inset. | we have updated our map following the reviewer’s comments. New map (Fig2) was plotted using R software. |
| Some references that might be useful: Dietrich K., et al. 2008. Integrated weight longlines with paired streamer lines – Best practice to prevent seabird bycatch in demersal longline fisheries. Biological Conservation. | this reference is now included in the Introduction and Discussion sections |

| **Reviewer 2 - Comments** | **Authors response** |
| --- | --- |
| This is an interesting study that address an important aspect when it comes to pinpointing reasons, and consequently also effective mitigation actions, for high bycatch rates in floated-demersal longlines in the north Atlantic. I think the results are highly relevant in a broader perspective, as it gives some general estimates to the sink rate in these types of fisheries, as well as shoeing the importance of weighted lines in reducing the time period when the bait is available for seabirds. The statistics/methods seems to appropriate, and I only have minor comments. Addressing them will probably remove my small concern about the seemingly very small N for the rather complex models used. Good work. | Thank you for this positive review |
| Title: Suggest to moderate the title, as it doesn’t seems like you have actually measured any changes in bycatch risk, although this is implied/assumed by the observed sink rate. | We agree and have updated the title as a result. New title is “Slow sink rate in floated demersal longline and implications for seabird bycatch risk” |
| Line 51: Would analysis be a better word than “data”, in “Recent data estimate that”? | Changed “data” to “publication” |
| Line 62: In my printed version it seems that there is a different font or color within this line, please check. | This has been corrected |
| Line 120-142: It is not clear to me whether all observation is done from one vessel, or if there are multiple vessels? Suggest to include this information in the text. | We have added a sentence in the *field work* methodology. |
| Line 141-142: It is a bit difficult to understand the rationale behind the different sections chosen as basis for the different models? Especially since there seems to be no focus on this in the discussion. Why overlapping intervals, from the text it seems to me that 0-2 (surface feeders) meters and 2-10 (diving) meter would be logical choices given the two different feeding regimes described? Suggest to clarify. | We have amended the last sentences of the Field work methodology, with “Those sections are 0 to 2m (to represent surface-feeders) and 0 to 10m (for pursuit-plunging divers) [Wiedenfeld 2016]. We also decided to include a 0 to 5m section to provide a more precise understanding of the bycatch risk across the water column. Analysis at 2 to 5m and 5 to 10m, will also help understand the sinking dynamic of the line at different depth sections and inform potential re-design of the gear.” |
| Line 161: How was it standardized, and why does this standardization ease interpretation? My initial though is that it would be easier to interpret according to actual setting speed and depth rather than a (mean?)standardized version of the variables for example, as I don’t know the range of the data. | We now specify how data was standardized. Data was z-transformed by mean centring and dividing by its standard deviation. Table 1 shows the range of the data each set. We think standardizing aids interpretability because it sets the intercept of our model at a meaningful value where 0 represents mean speed or depth rather than a water depth or speed of 0. We take the point that there is an advantage to looking at variables on their original scale as well, but the influence of speed or water depth on sink rate were secondary to our primary motivation which was the impact of net location and weighting and therefore we emphasized the interpretability of model intercept as far as we could. |
| Line 166: Not sure if I understand why predictive performance is in focus in the model selection, when the results seems to be predicted over mean’s (or the range, not explained) of all other variables than the TDR-positions. Why the model selection if all variables are relevant to account for when measuring effects on sink speed? Suggest to clarify. Also, I might have overlooked it, but I cant seem to find the results from the model selection? | Our primary motivation was to look at the effect of TDR location and how it was weighted. Therefore, the other variables, while interesting, were also things we wanted to remove if we could for model simplification purposes. This is because any predictions we make about sink rate from the models are conditioned upon whatever else is in that model. Therefore, if we can remove them and not say are results are conditional and x, y and z being held at set values we would prefer that. In other words, we thought it was advantageous to have the simplest model possible with the proviso it included information on TDR location.  The LOO CV does place an emphasis on the predictive performance of models. There are other approaches for model selection in a Bayesian context such as DIC which is provided by MCMCglmm outputs. The use of DIC has been questioned in some quarters, however. For us the use of LOO-CV was a simple method to develop and implement for model selection, so its use was primarily a pragmatic choice given that we did not want to use DIC. The LOO CV is now included within the Appendix, apologies for this previous oversight. |
| Line 168: would it be relevant to consider a vessel random effect as well? | See above. All data was collected from a single vessel across the duration of a fishing trip. Therefore, there was no opportunity to use vessel as a random effect. |
| Line 180: Change “used” to “using” | Corrected |
| 192: I don’t understand why a log transformation was used to prevent the model to predict negative values for depth. Or rather, I don’t understand why the model would predict negative values for depth if the proper distribution is assumed in the models. Did you assume a normal distribution, and is this assumption correct? Would a truncated distribution be a better choice? Suggest to include information about the type of model. | We chose to log-transform data prior to analysis because we wanted an approach that would prevent the prediction of negative values. In this case depth has a boundary of 0 so negative values would imply locations above the water. Therefore, we deliberately chose to model log-transformed data with this in mind. Another way of saying this would be to say depth was modelled using a log-normal distribution. i.e we thought a log-normal distribution (which is truncated at zero) was suitable / appropriate for this data. We never chose to employ a Gaussian model as we believed this would predict some negative values when TDRs are close to the surface.  Of course, other distributions that only allow positive values are possible including a truncated normal distribution, a gamma distribution etc. but log-normal models fitted well and are also more flexible (given you can log-transform the response variables), and there is abundant resources on how to back-transform values to the relevant scale exploiting the relationship between normal and log-normal variables. |
| Line 151-200: It’s a bit unclear to me why you chose to both construct models with sink rate as a response, and models with depth over time? Why not just the latter, as it seems to provide all the information (sink rates could easily be derived from these), and why not include TDR location as an interaction effect with time elapsed rather than creating 4 different models? Also, from the results it seems that you only have 91 lines in total (and much less per TDR location (?)), did you actually have enough statistical power to make sound estimate for all models/combinations. Your model-output seems to suggest that you have, but the model structure seems rather complex given the potential range for the different variable levels (e.g. as low as 6? for “middle”), and N in total? I have probably misunderstood this part, so please clarify in the text.   - *It’s a bit unclear to me why you chose to both construct models with sink rate as a response, and models with depth over time? :* - *why not include TDR location as an interaction effect with time elapsed rather than creating 4 different models :* - *from the results it seems that you only have 91 lines in total (and much less per TDR location (?)), did you actually have enough statistical power* | - The reasons for doing both are to provide alternative means at looking at sink rate for the development of policy alongside sink rates over predetermined depth ranges (e.g. 0-2 m, 2-5m etc.). Continuous models provide the most detailed picture of variation in sink rate over the course of a TDRs descent. However, our discussions with practitioners in this area and also colleagues responsible for developing policy is that sink rates over specified depth thresholds might be easier to work with. Here we wanted to provide a range of alternative ways of looking at sink rate. - Actually, this is more or less what we did. The model is set up with separate smoothers looking at sink rate versus time elapsed for each TDR position. i.e. a time by TDR position interaction. All the data is included within the same model framework, albeit we did estimate 4 smoothers of course and these are estimated with no pooling (independence) between different categories. So, the overall effect is, in the end, the same as if they were modelled in separate models. - In terms of power, we would need to know what specific tests were being considered and how big the effect we are looking for is. Larger effect sizes are obviously easier to detect reliably even with smaller sample sizes. For example, we claim in the results that: ‘TDRs at the weight reached 2 m, 5 m and 10 m much faster than those at other positions (Table 2).’ We believe the study has the requisite power to make this claim because the sink rate near weights is much greater than at other locations and we would expect this to represent a large effect size. This can be seen from the raw data plots but also is expected due to the nature of how weights should work. Another point of comparison we make is between estimated sink rates at different locations and the benchmark figure of 0.3 m / s. Here, we provide the results of a power analysis looking at the ability of our analysis to detect a range of different sizes when comparing our data to this fixed benchmark. Because the analysis presented in the main text that we are referring to here is a Bayesian analysis this requires adopting a slightly different approach to a power analysis - model simulation. To achieve this, we followed the simulation approach outlined recently in Elsey (2021). Briefly, we assumed a dataset in which there was a true underlying effect size of 0.01, 0.05,0.10,0.15, or 0.20. Note that effect sizes here were unstandardized so an effect size of 0.01 means we expect the true sinking rate to be 0.29 m / s versus a benchmark value of 0.3 m / s (i.e., a difference of 0.01 m / s). We also required some estimate of sampling variance, which we obtained as the standard deviation of our modelled estimates for sink rate. This means we can generate a number of simulated datasets with a ‘known’ underlying sink rate and associated sampling variance. In total we generated 250 simulations for each assumed effect size and then quantified the proportion of 95% CRI of models that spanned 0 to get an approximate indicator of statistical power. Results are presented below based on TDRs located on the middle of the net for which our sample size was lowest (and sampling variance was highest). Generally, we believe we have sufficient power to identify differences in sink rate of 0.05 m /s or greater versus a benchmark of 0.3. Though we would perhaps struggle to reliably detect a true difference of < 0.05 m / s at the middle position. Our power for detecting differences at other locations is expected to be greater than this as our sampling variance at these locations is less than at the middle. Depending on which tests the reviewer was referring to we can provide other tests of power, but would need to know which particular comparison or parameters was of most interest. For example, rather than looking at the difference between two estimates one could also look at 95% CRI interval width etc. Table 1. Results from power analysis looking at power to detect a range of different effect sizes based on a given sampling variance. Depth \| Location \| Sample Variance / SD(SD) \| Power over range of values for assumed effect size; 0 – 2 m \| Middle \| 0.014 \| 0.01 = 0.41 0.05 = 1.0 0.10 = 1.0 0.15 = 1.0 0.20 = 1.0; 2 – 5 m \| Middle \| 0.048 \| 0.01 = 0.08 0.05 = 0.64 0.10 = 0.99 0.15 = 1.0 0.20 = 1.0; 5 – 10 m \| Middle \| 0.037 \| 0.01 = 0.13 0.05 = 0.84 0.10 = 0.99 0.15 = 1.0 0.20 = 1.0 |
| Line 202: I miss a short description of which parameters comes into play in the different models (except for TDR-position, all seems to be omitted from the text (expect from being included in the sup. tables). It is thus difficult so see the rationale for doing a model selection at all, if these variables are of no interest. | We can add in a greater description of the predictor variables we found to be important in the results table. Ultimately, the only predictor that was retained in our models was wind speed assessed using the Beaufort scale. In this case wind speed was positively associated with the time taken to sink 0-5 metres (Table S4), 0 – 10 metres (Table S6) and 2-5 metres (Table S8). Likewise, wind speed was positively associated with the expected distance travelled from stern before TDRs reached 2 metre depth, 5 metre depth or 10 metre depth. These results suggest nets will tend to sink slower at higher speeds, though the size of any effect seems small (~3% - 7% depending on the exact model results table you are looking at).  Therefore, we add at the start of the results section the following paragraph:  ‘With the exception of wind speed, none of the predictor variables we initially included alongside TDR position were retained in our final models after our model selection procedure. Wind speed was found to be positively associated with the time taken to sink from 0 – 5 metres (Table S4), from 0 – 10 metres (Table S6) and from 2 – 5 metres (Table S8). Model coefficients suggest a one-unit increase on the Beaufort scale is predicted to lead to a small decrease in sink rate of ~ 3 -5%. Wind speed was also found to be positively associated with the expected distance travelled from stern before TDRs reached 2 metres (Table S14), 5 metres (Table S16), and 10 metres of depth (Table S18). Model coefficients suggest a one-unit increase on the Beaufort scale is predicted to lead to an increase in the distance travelled before reaching these depths as ~ 6 -8%.’ |
| Figures: Are these the predicted response across the range of all other variables, or the for example the mean? Please clarify. | We apologise but we are not entirely sure what figure is being referred to here. Boxplots in figure 4 are raw (unmodelled data. We have updated Fig 4 legend as “Box plots showing observed sink rates (raw, unmodelled data) obtained from TDR loggers at each TDR location across specified depth ranges. Red dotted lines indicate ACAP sinking speed recommendation (0.3 m / s).”  Figure 5 is based on GAMM outputs for which no other predictors were included. |

| **Reviewer 3 - Comments** | **Authors response** |
| --- | --- |
| This manuscript presents an assessment of the sinking speed of floated-demersal longline to determine potential bycatch risks to seabirds. First of all, I would like to present my congratulations to the author by this interesting and hard piece of work. Generally, the manuscript is well written and easy to understand. The main claim of the manuscript is well defined and properly placed in the context of the reviewed literature. However, despite data is likely to support the claims, some more details on the results are needed, namely in terms of the modelling exercises, how final models fit each sample and which predictor variables were included. At the end it was hard to find any detail on predicted variables other than TDR position or water depth, i.e. wind speed, setting speed and number of hooks. Such lack of details prevents the assessment of the appropriate and rigorous performance of the statistical analysis. Some details on GAMM results might be available under the supplementary material which as reviewer I had no access. Please find bellow my detailed revision: | Thank you for your review.  We have provided further information and details in the *Statistical Analysis* and *Results* sections of the manuscript, including predictor variables. More detailed results can be found in the Supplementary material. |
| Introduction Line 44 Consider to replace "Puffinus Gravis" by "Ardenna gravis" | corrected |
| Line 46 Replace "Celtic Sea" by "Celtic Seas" | corrected |
| Line 69 to 87 Consider to move this paragraph to a later section of your discussion. Here would be more interesting to introducing the reader to a narrow scope of your main topic/goal. | We understand the argument, but consider that giving some regulatory context in regard to seabird bycatch in similar demersal longline fisheries and in Europe, is important to properly introduce this fishery. It shows that in spite of efforts and regulation “on paper”, nothing tangible has happened to regulate or understand this fishery’s impact. And highlight the novel aspect of this paper. It provides context on known mitigation measures and in particular Bird Scaring Lines, which given our results are at the centre of our discussion at the end of the paper. |
| Methods Line 139 and 140 I found this affirmation too important to be based in such weak peaces of information reflecting a notable lack of data. Remembering the Gran Sol bycatch figures are based in very low observation effort, from only one vessel.  Despite, there are strong evidences of seabird bycatch to occur in the area, listing top bycatch species and bycatch numbers in the north east Atlantic floated demersal longline fishery based in such little information requires care. There is not much available information on how Great Shearwater bycatch rates were estimated for the Spanish fleet operating in Gran Sole, one important aspect to be into consideration is the presence of birds in the area during a narrow period of time. In the other hand, there are published evidences showing Northern Gannets mainly followed by Cory's Shearwater are being caught in serious numbers in longlines. Perhaps replace the assumption of "most bycaught species" by "two of the known bycaugh species" fits better the state of the art. Also, after reading the introduction and the methods I was expecting a need from the authors to discuss their findings in terms of any king of ecological or biological aspects of Fulmar or Great Shearwater. But, I understood those species are only used to justify the chosen distance bands. Perhaps, authors might be wider and justify their distance bands choice by the existence of different feeding strategies in seabird species. There are some classifications proposed by other authors, e.g. surface feeders, pelagic feeders, etc. And perhaps using Fulmar and Great Shearwater as an example of those groups, and add others if relevant. | The Gran Sol figures in Anderson et al. are derived from Spanish data collected across 37 fishing days and on two vessels. (10 days monitored in Autumn, 12 in Winter and 15 in Summer). G. Shearwaters represented ~60% of the birds bycaught, and Fulmars ~30%. In Northridge et al., figures are derived from 122 days at sea observed (0.86% of effort). Fulmars represented >92% of the catch. We believe that data from Anderson et al. and Northridge et al. are sufficient grounds to focus our argumentation on those two species, since according to the two sources, the combined G. Shearwaters & Fulmars represent over 90% of the bycatch composition.  Agreeing however that stating that Fulmars and G. Shearwaters are “[…] reportedly the most bycaught species [in this fishery]” might not reflect enough the data gaps that exist in this fishery. Paragraph has been changed to:  “We analysed the data according to the “bycatch risk depths” for Northern Fulmar and Great Shearwater, which have constituted over 90% of all bycaught birds in published studies of this fishery [3, 5], though it should be noted that overall this is a very data-poor fishery with regard to seabird bycatch. Other seabird species are being bycaught, though these are also primarily surface-feeding and pursuit-plunging species yet capable of deep diving [Ronconi et al. 2010, Garthe & Furness 2001]. We therefore split the water column into different depth sections to better understand the sinking dynamics of the gear in relation to the most-affected seabird’s feeding strategies [Ashmole et al. 1971].  Those sections are 0 to 2m (to represent surface-feeders) and 0 to 10m (for pursuit-plunging divers) [Wiedenfeld 2016]. We also decided to include a 0 to 5m section to provide a more precise understanding of the bycatch risk across the water column. Analysis at 2 to 5m and 5 to 10m, will also help understand the sinking dynamic of the line at different depth sections, and inform any potential re-design of the gear. |
| Line 160 Please clarify the choice of using log number of hooks instead the raw number. | We now state in methods: ‘We chose to model the number of hooks on the log scale as the raw number of hooks per net was typically very high with some discontinuity between values (range: 4000 – 10400) and we felt results would therefore be more interpretable on the log-scale.’ |
| Line 161 Please detail the standardized method used for setting speed and water depth | See response to reviewer above. We felt standardization in the way applied here (mean centring and dividing by standard deviation) made results more interpretable. Specifically, they may our model intercept and therefore estimates of sink at each TDR position more interpretable for us, particularly when we subsequently had to back-transform model predictions. However, we accept this is a matter of personal judgement more than anything. |
| Results Line 211 Table 1 - to confirm within the journal guidelines about measurement units, knots are not an IS unit; Perhaps it is worth to remove "Species targeted", because all samples were targeting hake. | The knot is a non-SI unit accepted for use with the International System of Units (SI) [ref: JCGM 200:2012]. Given the conservation and fishing focus of the paper, we also consider the use of knots to define vessel’s speed, to be more in tune with the practice of the fishing industry/ Column “species targeted” has beem removed. |
| 214 What about the results of the modelling? How much the predictor variables explain the sink rate variability? Which predictor variables were taking into account in the final model? Only the effect of position of TDR on the line on sink rates are given. Perhaps the results of the LOO CV step to retain or remove predictors are missing, but important to clarify the authors' choices. More details on those results are needed. | The top performing models in each case are presented in the supplementary material allowing one to see which predictors were retained alongside net position. We now also write a paragraph at the start of the results describing which predictors were retained (only wind speed in the end) with some interpretation of this before moving on to focus on the influence of TDR location on the net.  We have also added an estimate of the R^2^ for each of or models as well so one can see how much variation they explain. In general, our R^2^ is very high, because TDR net location is a strong driver of variation in sinking rate (as expected of course). Note for Bayesian models we can get R^2^ with 95% CRI as well.  Details on model selection via the LOO-CV process are also now added to the Supplementary Material. |
| Line 237 As stating before, apart from the outputs of GAMM modelling, the results of the modelling exercise itself will help reader to understand the goodness of fit of the selected model. | note that for the GAMM modelling no model selection was conducted. The model includes separate smoothers for TDR positions. Given our primary goal was to investigate sink rate at these positions we did not feel to need to examine whether or not this improved model fit, we simply wanted to know what these different smoothers looked like and report them. The model results and coefficients themselves are displayed in the Supplementary Material however. |
| Line 255 It is a very non-important detail, but as sink rate is expectable better when above 0.3m/s my suggestion is to illustrate it with a black line, and when bellow as a red line. | Colours changed as suggested. |
| Line 262 Still this paragraph about sink rate or more about "Distance from stern for specific water depth ranges". Consider to add this "sub-chapter" here or follow the same approach used in the Methods - "Sink Rate and Distance from..." - for the entire sub-chapter | Added sub-paragraph title “Distance from vessel and hooks sinking dynamics” |
| 266 Authors use knots, please confirm guidelines for the need of using IS units. | See above answer |
| Discussion Line 275 to 324 Depending on the results, it would be worth to discuss the possible effects of wind speed, setting speed and number of hooks (as proxy for size of a longline) on the sink rates and then on the bycatch risk. | added paragraph “Although environmental factors were recorded during field work, we were not able to assess their influence on sinking speed. Wind speed has shown a positive relationship with hook sink rate in a drifting-pelagic longline fishery [Anderson & Mcardle 2002], but the drastically different weighting regime renders any assumptions hazardous in our case. Slower vessel setting speed is also known to reduce the amount of time hooks remain at the surface and at depths that seabirds can reach [Wanless & Waugh 2010], although its effect on a floated-demersal longline and the practicality of reduced vessel speed during fishing operations needs to be investigated”. |
| Also, only one vessel was sampled in this study. Could such fact be a limitation for this study? Might be desired to discuss it in the light of the variability of longlines even within the same fishing fleet. | We agree with the reviewer that data sourced from only one longliner is a limit of this study. This was mainly due to limited funding and difficulties in securing collaboration with volunteer vessels within the fleet. We have however included further information and references in regard to the gear’s configuration representativeness within the fleet – see *Field Work Methodology* section.  We also discussed in more details potential effects of environmental factors, in the *Results* section. |
| Lines 313 to 316 Could authors discuss the main challenges for fishers regarding changes in the longline weighting? | added sentence in the second last paragraph “For instance, a switch from current 3kg to 5kg weights for a set of ten thousand hooks would represent roughly an additional half a tonne to be carried onboard and handled by fishers.” |

| **Reviewer 4- Comments** | **Authors response** |
| --- | --- |
| General issues: Rouxel et al. present a method to assess the sink rate of a floated-demersal longline, and apply this method to a longline used to target European Hake in the offshore waters of Scotland, and find that the sink rate is slower than the rate recommended by the ACAP, in the top 2m of the water column in particular, and the distance astern of the vessel for hooks to sink beyond seabirds’ reach largely exceeds the optimum coverage. They indicate that hooks from floated-demersal longlines present a clear bycatch risk. I found the experimental method adequately motivated, and I believe it will be useful in the following seabird bycatch analyses. I have some concerns about the sufficiency of an experiment on a single longline to achieve a general conclusion. Moreover, there is no observation on seabird bycatch during this experiment, and no analysis on the impacts of sink rate and other factors on seabird bycatch, so it is a hypothesis that the slow sink rate in this floated-demersal longline may pose high bycatch risk to seabirds, but to what extent, we don’t know. I imagine that this kind of fundamental experiment will be useful if a following analysis is conducted to relate seabird bycatch to the sink rate. At this point, I suggest to revise the title to avoid overstating this study and add more references in the discussion to emphasize the potential impacts of sink rate on seabird bycatch. All concerns should be addressed via major revision. | Answer: We agree with the reviewer that follow up analysis is needed to properly understand the direct relationship between sinking speed of floated-demersal longlines and seabird bycatch. However, we consider that the high bycatch rates/estimates observed in Anderson et al. (2011) and Northridge et al. (2020) for the Gran sol fishery strongly suggests the particularly risky nature of this gear for seabirds. Our results show a very slow sink rate and are in line with publications and requirements under the Agreement for the Conservation of Albatrosses and Petrels (ACAP), which directly link slow sink rate to high bycatch risks for seabirds. To reflect the remaining uncertainties pointed out by the reviewer however, we decided to rephrase the paper’s title to: “Slow sink rate in floated-demersal longlines and implications for seabird bycatch risk”. We also provided answers to the other points, with corrections as follow. |
| Specific issues: Lines 41-43: Where were these numbers (160,000-320,000) from? | from Anderson et al. 2011. Reference was provided on the following sentence, but now moved to the end of this sentence, for better clarity. |
| Line 76: Please specify the best practice mitigation measures. | those are night setting, Bird-scaring lines, or line weighting, and are listed in the previous sentence. “Best practice” now removed and replaced by “those aforementioned” for better clarity. |
| Lines 125-129: Did weight keep constant? | we address this question at line 96, “[…] 3kg weights spaced every 100m appears as a common configuration in this fishery (Figure 1).”, and at lines 128-129 “these trials were conducted on the most common configuration of the gear”. For better clarity however and supportive references, we added “[…] according to the fishery observer, and echoes the configuration recorded in other semi-pelagic Hake fisheries across the Atlantic [Paterson et al. 2019, Petersen et al. 2008, Erzini et al. 2001]”.  Weights are “off the shelf” and handmade small concrete blocks, and weigh roughly ~3kg. Given the non-standardisation of this equipment, it is hard to tell if all those weights are constant between vessels. Nevertheless, two different skippers and one experienced observer in this fleet confirmed that 3kg weights are common practice. |
| Lines 158-160: Does other factors, such as wind direction, wave height, weight (if constant during this experimental period), influence the sink rate? | the list of predictors we examined were those that were recorded by the observer upon net deployment. We did not consider other variables |
| Lines 182-183: It was found that reducing setting speed reduced line tension and resulted in gear sinking closer to the vessel (Pierre et al. 2013). Is it the case in this study? | This study looked at sinking speed of the gear during normal fishing conditions, and fishers were asked to operate as usual. In the absence of pre-existing data on this fishery, the idea was to provide a baseline. We hope to respond to such question in follow up studies.  We however added a paragraph in the discussion section “Slower vessel setting speed is also known to reduce the amount of time hooks remain at the surface and at depths that seabirds can reach [Wanless & Waugh 2010], although its effect on a floated-demersal longline and the practicality of reduced vessel speed during fishing operations needs to be investigated.” |
| Lines 288-289: Need references. | we deleted the following: “and as a result are easily available to the widest array of seabird species, including Northern Fulmars, the species considered to be most impacted by this fishing gear in this location” |
| Lines 312-324: There have been sufficient studies investigating the impacts of sink rate of longline fisheries on seabird bycatch (e.g. Cortés et al. 2018). Methods such as weighted lines, Chilean system, thawing the bait, smaller distance between consecutive weights have been adopted to increase sink rate in order to reduce seabird bycatch, although these methods have been found with some operational problems. For example, an increase of entanglements between the branch lines and hooks may happen during the setting operations when additional weights were used. More discussion on how to increase sink rate and the corresponding impacts on fish catch will improve this paper. | We have added in the second last paragraph under Discussion section the following: “For instance, a switch from current 3kg to 5kg weights for a set of ten thousand hooks would represent roughly an additional half a tonne to be carried onboard and handled by fishers. Cortés & González-Solís [XX] also found that in artisanal demersal longlines, more weight is susceptible to increase entanglement risks between the branchlines and hooks during the setting operations. However, change in the longline weighting regime is the single measure most likely to deliver a significant reduction in seabird bycatch, and a series of measures that could help reduce operational issues should be investigated such as alternative spacing between branchlines and between weights [Jiménez et al. 2013], use of steel weights instead of concrete [Paterson et al. 2019], etc. When paired with bird scaring lines, appropriate weighting regime can virtually eliminate seabird bycatch from demersal longline fisheries [Dietrich et al. 2008]. “  We consider however that deeper discussion on how to increase sink rate and the corresponding impacts on fish catch – while absolutely necessary to drive change in this fishery - would be better suited for a follow up study and paper. In particular regarding impact on fish catch (see below answer) |
| A study found that weighted lines increased blackmouth catshark catches, possibly because catsharks forages in the near bottom layer and on the seabed (Anastasopoulou et al. 2013). An introduction on the life history of the target species European Hake would help to discuss the potential impacts of increased sink rate. | European Hakes are demersal fish species found in waters 70-350 m deep. To target this species, the longline need to reach sufficient depth for its weights to hit the seabed and floats to lift the line where hooks are attached. Increasing the weight at current sinkers places (e.g. switching from 3 to 5kg) would certainly increase sink rate, but isn’t expected to drastically change the dynamic of the gear when fully set. We feel that introducing life history description of the target species, to explore potential impacts on catch rate derived from structural changes on the gear, isn’t supported by enough information at this stage and requires dedicated research. We believe would not improve the impact of the paper. However, we would like to address this question in a follow up study and paper. |
| Figures 4 and 5: The four panels for each figure can be put in one page, using some R packages such as ggplot2. | at the moment plots are supplied separately because in the past we have found it easier / more flexible to submit panelled figures in this way prior to publication. Plots can be put on a single page for publication after discussion with journal editors if required. |

| **Reviewer 5- Comments** | **Authors response** |
| --- | --- |
| This is an excellent manuscript and was a pleasure to review. I have a few minor suggestions for consideration:  - line 83. Provide a reference for 'Clean Catch UK' | we have added the following Reference:  Hetherington, S.J., Bendall, V.A., Wild, M., Clare, T., Ryan, K., Ashton, J., Engelhard, G., Bell, C., Day, E., Kelman, E. (2021). Clean Catch UK: Joint Action to Reduce Wildlife Bycatch, Annual Report (April 2020 – March 2021). 11 pp. |
| - consider combining Figures 1 and 3 | While we agree that combining Fig.1 and Fig. 3 would be interesting, we believe important to keep representations of the gears’ configuration (Fig1) and TDRs positions (Fig3) split as to keep figures as clear and comprehensive as possible. Fig.1 provides for the first time in the published literature detailed gear configuration in this fleet, and as such we believe important to keep it separated from our project’s “influence”. |
| - lines 130-132. Consider rewriting as follows: Fishers were asked to deploy gear as they would in a normal fishing operation. Eight to ten TDRs were deployed on each experimental set, depending on fishing conditions. | accepted suggestion. |
| - line 139. Delete 'reportedly the most bycaught species in the north east Atlantic floated demersal longline fishery', as this repeats information provided earlier. | deleted suggestion. |
| - line 182. insert 'that' between stern and different, i.e., ..... from the stern that different positions .... | accepted suggestion. |
| - line 255. Spelling - polygon. | corrected |
| - reference 41. Please provide more details, e.g., name of university. | we have completed the reference as follow:  Kühn, S. (2016). Loss of longline-bait to northern fulmars: economic balance between damage from bait-loss and costs of measures to reduce seabird bycatch on the Faroe Islands (Doctoral dissertation, University of Akureyri, Ísafjörður, Iceland). Retrieved from <http://hdl.handle.net/1946/25479> |
