# Supplementary material for: Slow sink rate in floated-demersal longline and implications for seabird bycatch risk
Source: PLoS One. 2022 Apr 28;17(4):e0267169. doi: 10.1371/journal.pone.0267169 (PMC9049334; doi:10.1371/journal.pone.0267169)
Supplement: Attachment — Submitted filename: Response to Reviewers_Rouxel et al_2V.docx [file pone.0267169.s020.docx]

Response to reviewers:

| **Reviewer 1 - Comments** | **Authors response** |
| --- | --- |
| The authors have done a good job revising the manuscript. That some of the environmental variables not showing up as significant as we thought they would be in some of the models may be because a single set of covariates were recorded for each longline set, which includes multiple TDR tracks (see Table 1), and the relatively narrow geographical extent (Fig 2), thus limiting the contrast in the recorded covariates. Extended monitoring involving multiple vessels operating across different seasons may be needed in further work, preferably with concurrent catch and bycatch monitoring. Nonetheless, the result is clear that the sink rate at non-weighted sections is substantially slower than the ACAP recommendation.  I have just some minor editorial comments:  1, Please conform to journal guidelines on tables. "Tables must be editable, cell-based objects" instead of images. | Tables within the Manuscript (word document) are editable and not images. |

| **Reviewer 3 - Comments** | **Authors response** |
| --- | --- |
| Thank you for addressing all comments. I only add a couple of minor suggestions: - line 228 - later in line 384, a specific reference is given in a different format, i.e. Cortés & González-Solís [51]. Please double check journal guidelines. | Guidelines indicate *“In the text, cite the reference number in square brackets (e.g., “We used the techniques developed by our colleagues [19] to analyze the data”). PLOS uses the numbered citation (citation-sequence) method and first six authors, et al.”*  For consistency, we removed year reference in line 228. |
| line 266 - might be more clear if the specific tables were listed. | While we agree citing specific tables could help, the fact that nearly half of the Tables within the Supporting Information include predictor variables – eight tables out of eighteen –, we believe it would make the citation very long and not particularly making it clearer to the reader. |

| **Reviewer 4 - Comments** | **Authors response** |
| --- | --- |
| The manuscript “Slow sink rate in floated-demersal longline and implications for seabird bycatch risk” was resubmitted with extensive revisions. The authors have effectively responded to the various comments and suggestions and the manuscript is much improved. In particular, I found the extensive revisions of the discussion very helpful, as well as improved descriptions of models used. I only have a few minor comments as follows:  Lines 156-157: Merge to the last paragraph. It is weird for a sentence to be a paragraph. | Contrary to what the tracked change view in the “Revised Manuscript” looks like, the sentence mentioned is part of a wider paragraph. Please check the “cleaned” version of the Manuscript. |
| Line 225: There is no need to state the full name of credible intervals, because it has appeared before in Line 197. | We amended the sentence as follow “*Because we are using Bayesian models sink rates can be calculated across model posterior distributions allowing easy calculation of 95% CRI”.* We decided to keep the sentence itself in however, because it relates to how *Posterior distributions* can be used to generate calculated estimates like sink speed, etc. |
| Line 234: Change “is” to “was”. | Corrected |
| Lines 660-661: Add journal information: New Zealand Journal of Marine and Freshwater Research, 36(1), 185-195, DOI: 10.1080/00288330.2002.9517079. | Added |
